# The Beat

**DOI:** 10.1289/ehp.120-a20b

**Published:** 2012-01-01

**Authors:** Erin E. Dooley

## Rotating Shift Work and Type 2 Diabetes in Women

In a prospective study of more than 175,000 U.S. women, those who worked a rotating schedule that included 3 or more night shifts per month had a higher risk of developing type 2 diabetes than women who worked only days or only nights.^1^ Women who worked rotating shifts were also more likely than other women to become obese during the 18–20 years of followup. The results suggest the increased risk of diabetes may be at least partly mediated through weight gain.

## NICEATM and ICCVAM Announce Request for Comments

In November 2011 the National Institute of Environmental Health Sciences and the National Toxicology Program Interagency Center for the Evaluation of Alternative Toxicological Methods (NICEATM) issued a request for comments to be considered by NICEATM and the Interagency Coordinating Committee on the Validation of Alternative Methods (ICCVAM) in updating the current NICEATM/ICCVAM 5-year plan to cover the years 2013–2017.^2^ ICCVAM was established as a permanent committee in 2000 to promote the development, validation, and regulatory acceptance of new or revised alternative toxicological test methods that reduce, refine, or replacing animal tests while ensuring human safety and product effectiveness. Interested parties should submit their comments by 15 January 2012.

## Landmark Climate Gains in Durban

In December 2011 the 194-party United Nations Framework Convention on Climate Change reached agreement on a complex and overarching program aimed at setting a new course for worldwide action against climate change.^3^ The parties agreed to negotiate a legally binding agreement by 2015 to come into force by 2020, which would apply to developed and developing nations alike. They also made progress toward establishing a Green Climate Fund to administer billions of dollars for climate change adaptation measures in developing nations as well as rules for protecting and conserving forests.

## Lead Battery Recycling on the Rise in Mexico

A recent investigation by *The New York Times* found that about 20% of spent U.S. vehicle and industrial batteries end up in Mexico, up from 6% in 2007, where weaker environmental standards and lax enforcement jeopardize communities near recycling plants.^4^ Spent batteries can contain up to 40 pounds of lead, which can be released as dust or fumes during the recycling process. Advocacy groups are calling on U.S. companies to export spent batteries only to countries whose standards are as strict as those in the United States.

## New Tool Maps Pollution, Noise Levels across Europe

In December 2011 the European Environment Agency (EEA), Microsoft, and technology company Esri launched the Eye on Earth network, a “cloud computing” platform that maps air, water, and noise pollution across Europe based on government data and information uploaded by users.^5^ The WaterWatch feature displays 22,000 locations where the EEA monitors water quality, AirWatch provides information on more than 1,000 air-monitoring stations, and NoiseWatch allows users to directly upload noise-level readings from their personal mobile devices.

“The vision of the Eye on Earth is to help communities around the world get easier and more timely access to environmental and societal data and information they need to design and create a future that is sustainable and resilient to the changes and challenges ahead,” says EEA executive director Jacqueline McGlade. Additional services are planned for the network over the next five years.
